# Aging dimensions and markers as relative predictors of mortality in a longitudinal epidemiological sample

**DOI:** 10.1371/journal.pone.0324156

**Published:** 2025-06-18

**Authors:** Kristian E. Markon, Frank D. Mann, Colin D. Freilich, Steve Cole, Robert F. Krueger

**Affiliations:** 1 University of Minnesota, Minneapolis, Minnesota, United States of America; 2 Stony Brook Medicine, Stony Brook, New York, USA,; 3 UCLA, Los Angeles, California; Rutgers The State University of New Jersey, UNITED STATES OF AMERICA

## Abstract

Measurement of aging is critical to understanding its causes and developing interventions, but little consensus exists on what components such measurements should include or how they perform in predicting mortality. The aim of this study was to identify factors of aging among a comprehensive set of indicators, and to evaluate their relative performance in predicting mortality. Measurements on 34 clinical, survey, and neuroimaging variables, along with epigenetic age markers, were obtained from two waves (2004–2021) of the Midlife in the United States (MIDUS) study. Mortality data was also available on 11875 participants, including 1908 twins. Factor analyses were used to identify aging factors, and these were used to predict mortality as of 2022. Twin data were used to model predictors of mortality within families. Factor analyses identified 9 major dimensions of aging: frailty, cognition, adiposity, glucose, blood pressure, inflammation, lipids, adaptive functioning, and neurological functioning. The strongest predictors of survival among the aging dimensions were cognition, adaptive functioning, and inflammation, and among the epigenetic markers, the decline-predictive markers (GrimAge and DunedinPACE). When entered in joint prediction models, cognition remained a significant predictor of mortality, but the epigenetic markers did not. Cognition, adaptive functioning, and inflammation remained significant predictors of mortality within twin pairs as well. Aging is a multidimensional construct, with cognition, adaptive functioning, and inflammation being the strongest predictors of survival among the aging dimensions examined. Their association with mortality is observed within families, suggesting that early developmental factors cannot entirely account for their association with survival. Interventions and assessments should prioritize cognition in measures of aging quality, along with adaptive functioning and inflammation.

## Introduction

Characterizing the aging process and its relationship with an endpoint in mortality is critically important, especially with populations increasing in age worldwide [1]. Various markers have been proposed as indices of quality and rate of aging, but how these markers are empirically organized into aging factors is poorly understood, and there is substantial disagreement about what constitutes the aging process, in terms of its essential features, its causes, and how it should be measured [2]. Relatedly, many widely used aging markers (such as epigenetic aging markers) were developed with aging conceptualized as a single dimension of biological or chronological age [3–5]. However, it is possible, if not likely, that aging is characterized differently in different individuals, and that different biological, behavioral, and health-related markers of aging might relate differently across various dimensions of aging.

Another key question is when in the lifespan aging markers first come to be associated with accelerated age and mortality. Early environmental factors, for instance, have been linked to accelerated aging later in life [6], as have genetic factors [7]. Delineating when the relationship between aging markers and later mortality first manifests is important for understanding etiology and designing interventions, both in terms of determining the optimal timing for intervention and highlighting specific processes to target.

Here, we aimed to identify the empirical structure of markers that have been used as, or proposed for, general aging markers, to guide future research on aging processes, to examine their relationships with epigenetic markers of aging, and to examine their ability to predict mortality in a longitudinal epidemiological US sample containing a unique breadth of survey, clinical, and biological measures of aging. We also examined associations between aging markers and mortality within twin pairs to distinguish between associations stemming from genetic or environmental factors shared within families from those unique to individuals, which often emerge later in life.

## Methods

### Sample and design

Participants were those from the Midlife Development in the United States (MIDUS) study with mortality data as of 2022 (N = 11875). Analyses focused on two waves of data collection in which biological and clinical data were collected: Waves 2 and 3 of the MIDUS Core sample, and Wave 1 of the Refresher cohort, which was designed to replicate the Core sample in a new cohort. For the purposes of the current analyses, the Wave 3 Core and Wave 1 Refresher cohort were combined, as they were collected in overlapping years (2013–2021 and 2011–2015, respectively), were more proximal to the time of the mortality data collection, and had similar variables available that were not available in the Core Wave 2 collection (e.g., certain balance measures and neurobiological variables). Throughout this manuscript, the Core 2 wave and the combined Core 3 and Refresher wave, are referred to as Time 1 and Time 2 respectively.

Participants also included 1908 twins — 715 monozygotic (MZ) twins and 1193 dizygotic (DZ) twins, with 874 complete pairs, 341 MZ pairs, and 533 DZ pairs. Characteristics of the overall sample are presented in Table S1 in S1 File; Details regarding MIDUS are also available at the MIDUS website and in other publications (https://www.midus.wisc.edu).

### Selection and factor analysis of aging variables

Aging variables were selected because they have appeared in previous papers on the structure of aging indicators [8–11], they have been used as indicators of general aging [3], or they have been recommended as aging indicators in the review or protocol literature [12,13]. Four variables were available during Time 2 that were not available at Time 1: two measures of balance (Romberg sway path area and path length), hearing acuity, and a measure of brain age [14]. Further details regarding the measures are described in the supplement.

Variables were modeled using exploratory factor analysis (EFA) with full information maximum likelihood (FIML) estimation for the full set of indicators, followed by confirmatory factor analysis (CFA) to examine the fit of aging models used to construct factor indicators as used in predictive models. Structural models were fit to data from both waves and replicability across waves was used as a criterion for model and indicator retention.

### Epigenetic aging markers

Fasting blood draws were obtained from the MIDUS Core sample from 2004 to 2009 and from the MIDUS Refresher sample from 2012 to 2016. Whole blood samples were collected using a BD Vacutainer Tube with EDTA anticoagulant and frozen in storage. In 2019, DNA methylation profiling was conducted on the whole blood DNA samples from both the Core and Refresher samples. After DNA was tested for suitable yield and integrity, it was subjected to genome-wide methylation profiling using Illumina Methylation EPIC microarrays. The resulting beta values were noob-normalized to control for technical sources of variance, registered onto the list of CpG sites assayed on the Illumina Methylation 450K microarray, and screened using standard quality control metrics. Raw methylation data was used to score the following markers: Horvath [4], Hannum [5], PhenoAge [15], GrimAge Version 2 [16], and DunedinPACE epigenetic pace of aging markers [3]. The first two markers, sometimes referred to as first generation markers, were developed by identifying methylation sites predictive of chronological age; the second set of two, sometimes referred to as second generation markers, were developed using sites predictive of health and mortality variables; DunedinPACE, sometimes referred to as a second or third generation marker, was developed via methylation sites predictive of aging-related health decline. Epigenetic age acceleration by extension is defined as the residual of an individual’s value on an aging marker from their predicted chronological age. In addition, we examined State and Decline factors as summaries of the markers, with the first three epigenetic age acceleration (EAA) markers being used to construct a state factor score (i.e., Thurstone regression score), and the others being used to construct a decline score, reflecting declining health including its extreme form in death [17]. More information on the collection and the derivation of epigenetic variables in MIDUS is available in the data documentation on the MIDUS Colectica Portal (https://midus.colectica.org/).

### Mortality and its prediction

Aging factor indicators identified in the first step were used together with EAA markers to predict mortality. Survival was predicted using logistic regression [18], with survival status at 2022 as a binary outcome variable (coded such that survival = 1 and mortality = 0). Cox proportional hazard models were also attempted, but these did not always converge in estimation and were not available in the software for use in within-twin-pair models. Two sets of predictive analyses were conducted, one using all available data with twin family treated as random effect; another set of analyses were conducted only using twins, modeling within-pair effects in a mixed effect model. In prediction models, all variables were adjusted for chronological age, sex, ethnicity, and cohort. Estimation was completed using maximum likelihood with full information treatment of missingness, with robust information matrices and standard errors, in Mplus 8.10.

## Results

### Factor analyses of aging variables

Parallel analyses (using the 95th percentile of random eigenvalues as a threshold) suggested a best-fitting model with 10 factors for Time 1 and 11 factors for Time 2. In the 11-factor EFA models (Tables S12-S13 in S1 File), 9 of the factors replicated across waves: frailty, memory, executive function, adaptive function, adiposity, glucose, blood pressure, lipids, and inflammation. The factors unique to each wave included single measures and another factor: BMI and perceived health at Time 1, and brain age and vestibular function, which were not measured at Time 1, at Time 2. The 11-factor replicated factors were also present in the 10-factor model estimates (Tables S10-S11 in S1 File), with the exception of perceived health at Time 1 and lipids at Time 2. Given the closely related content of the cognitive factors, models with fewer factors were also fit (Tables S2-S9 in S1 File). In these factors the vestibular and neurological markers, and the memory and executive function factors, merged to form superordinate neurological function and cognitive factors.

Given the replicability of the factors across waves and models, the close relationships between the neurological and cognitive subfactors, the parallel analysis results, and to avoid single-indicator factors, an 8/9-factor model (9 with one factor not represented at Time 1) was fit using CFA. This model included factors reflecting frailty (or reversed as strength; with indicators including grip strength, peak flow, bone mineral density, and waist-hip ratio), cognition (BTACT), adiposity (BMI, percent lean mass, CRP), blood glucose (fasting glucose and HbA1c), blood pressure (systolic and diastolic), inflammation (IL8, IL10, and TNF), blood lipids (total cholesterol and triglycerides), adaptive functioning (ADL score, chair stand task, timed walk task, and self-rated health), and neurological functioning (Cole brain age, hearing acuity, and Romberg balance). This model fit acceptably (Table S14 in S1 File), and better than a model with a single aging factor. A bifactor model with 9 specific factors and a superordinate general aging factor fit better, but in that model, cognitive measures were split again into working memory and memory components across the general and specific factors, respectively (Table S14 in S1 File); a model with that split but no superordinate factor fit best, raising questions about the nature of the general factor in the bifactor model (Table S15 in S1 File). We ultimately chose to use the 8/9 factor model for predictive analyses for two reasons: first, because the 9/10-factor model differed from the 8/9-factor model only in splitting indicators from the same measure into two subfactors, with the possibility of one (working memory, with its two indicators coming from the same subtest) reflecting a test method effect; and second, because of interpretive ambiguities about the nature of the general factor in the bifactor model. For predictive models, sum scores corresponding to each of the factors were created by summing centered and scaled markers of each factor; sum scores were used rather than other factor score estimates to minimize correlations due to cross-loadings, and to minimize error due to factor loading estimation error [19,20].

### Mortality prediction and associations with epigenetic markers

#### Overall sample.

Regression results for the overall sample, predicting survival, are presented in Tables 1-2. The strongest predictions of survival across the two waves were cognition (β = 0.09 and 0.23 at T1 and T2), inflammation (β = −0.09 and −0.46), and adaptive functioning (β = −0.20 and −0.27), which were significantly predictive of survival at both time points, as well as on average (β = 0.14, −0.24, and −0.24, respectively). Strength was significantly predictive at both time points, but the average across timepoints was not significant. Blood glucose was significantly predictive only at T1.

**Table 1:** Prediction of Survival from Aging Factors: Overall Sample.

	N^*^	R^2^	β	se(β)	p	q
**Mean**						
Strength	684	0.00	−0.06	0.08	0.40	0.46
Cognition	988	0.02	0.14	0.05	< 0.001	0.01
Adiposity	1063	0.00	−0.01	0.05	0.80	0.80
Blood Glucose	678	0.02	−0.13	0.06	< 0.001	0.01
Blood Pressure	686	0.02	0.13	0.09	0.25	0.33
Inflammation	677	0.06	−0.24	0.10	< 0.001	< 0.001
Blood lipids	677	0.02	0.14	0.10	0.22	0.33
Adaptive Functioning (-)	1130	0.06	−0.24	0.04	< 0.001	< 0.001
**T1**						
Strength	1253	0.01	0.12	0.04	< 0.001	0.01
Cognition	1152	0.01	0.09	0.04	0.02	0.04
Adiposity	1255	0.00	−0.05	0.04	0.21	0.28
Blood Glucose	1242	0.01	−0.08	0.03	< 0.001	< 0.001
Blood Pressure	1253	0.00	−0.02	0.04	0.57	0.65
Inflammation	1240	0.01	−0.09	0.04	0.01	0.02
Blood lipids	1243	0.00	−0.01	0.05	0.85	0.85
Adaptive Functioning (-)	1255	0.04	−0.20	0.04	< 0.001	< 0.001
**T2**						
Strength	1422	0.04	0.20	0.08	0.02	0.03
Cognition	1774	0.06	0.23	0.04	< 0.001	< 0.001
Adiposity	1804	0.00	0.01	0.05	0.93	0.93
Blood Glucose	1415	0.00	−0.03	0.05	0.42	0.54
Blood Pressure	1428	0.01	0.09	0.08	0.27	0.40
Inflammation	1415	0.21	−0.46	0.11	< 0.001	< 0.001
Blood lipids	1414	0.00	−0.01	0.07	0.79	0.89
Adaptive Functioning (-)	1871	0.07	−0.27	0.04	< 0.001	< 0.001
Neurological Functioning	495	0.01	−0.09	0.08	0.01	0.01

Values are sample size for the predictor values (N^*^; note that the N for the outcome variable is 11875 in all cases), R^2^, standardized slope (β), standard error of the slope, p-value, and q-value.

**Table 2:** Prediction of Survival from Epigenetic Variables: Overall Sample.

	N^*^	R^2^	β	se(β)	p	q
Horvath	1200	0.00	0.00	0.07	> 0.99	>0.99
Hannum	1200	0.00	−0.05	0.06	0.32	0.45
PhenoAge	1200	0.02	−0.12	0.05	0.01	0.02
GrimAge	1199	0.07	−0.26	0.04	< 0.001	< 0.001
DunedinPACE	1200	0.08	−0.29	0.04	< 0.001	< 0.001
State Factor	1199	0.00	−0.04	0.07	0.52	0.61
Decline Factor	1199	0.08	−0.29	0.04	< 0.001	< 0.001

Values are sampl\e size for the predictor values (N^*^; note that the N for the outcome variable is 11875 in all cases), R^2^, standardized slope (β), standard error of the slope, p-value, and q-value.

**Fig 1:**
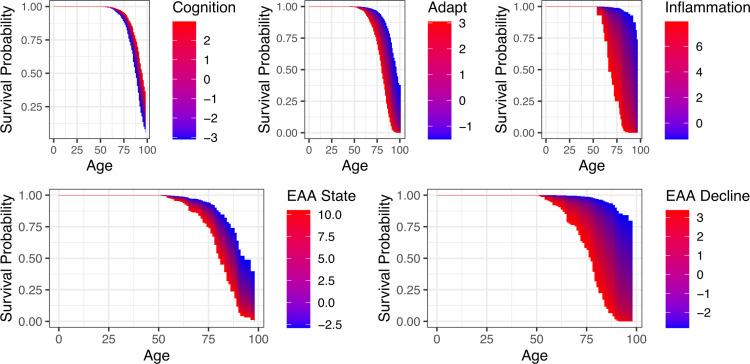
Survival probability as a function of age and predictors (aging factors and epigenetic markers). Survival curve gradients are shown for cognition, adaptive functioning, inflammation, and epigenetic age acceleration state-predictive factor and decline-predictive factor in the overall sample, moving from left to right and top to bottom. Gradients are shown with regard to the standardized predictor value averaged over the two waves.

Among the epigenetic age acceleration variables, all predictors were significantly predictive of survival except the Horvath and Hannum markers. The two decline markers, GrimAge and DunedinPACE, were more strongly predictive of survival than PhenoAge, and the difference between the state and decline markers was reflected in the respective factors, where the decline factor was significantly predictive of survival but the state factor was not (survival curves are illustrated in Fig 1 for the two epigenetic age acceleration factors and cognition and adaptive functioning).

Correlations between the aging factors and epigenetic markers are given in Table S16 in S1 File. Cognition and adaptive functioning were both significantly correlated with GrimAge and DunedinPACE at both waves (r = −0.107 to −0.148 for cognition, and 0.157 to 0.185 for adaptive functioning), adiposity was correlated with DunedinPACE and PhenoAge at both waves (r = 0.163 to 0.315), inflammation was correlated with Hannum and PhenoAge at both waves (r = 0.054 to 0.097), and blood glucose was correlated with DunedinPACE at both waves (r = 0.066 and 0.136). Other correlations between aging factors and epigenetic markers did not replicate across waves.

The relative ability of the aging factors and epigenetic markers to predict mortality was examined by including the aging factors and epigenetic markers that significantly predicted mortality at both T1 and T2 (strength, cognition, adaptive functioning, inflammation, PhenoAge, GrimAge, and DunedinPACE) together in a prediction model (Table 3). Cognition predicted survival at both waves (β = −0.24 and 0.29; i.e., improvements in cognition predicted greater probability of survival) and adaptive functioning predicted survival at T2 (β = −0.13), although this did not remain statistically significant after correction for multiple testing. Strength did not significantly predict survival in the presence of the other predictors, nor did any of the epigenetic markers.

**Table 3:** Prediction of Survival from Aging Factors and Epigenetic Variables: Overall Sample.

	N^*^	β	se(β)	p	q
**T1**					
Strength	1253	0.03	0.08	0.68	0.75
Cognition	1152	−0.24	0.06	< 0.001	< 0.001
Adaptive Functioning (-)	1255	−0.03	0.05	0.53	0.64
Inflammation	1240	0.00	0.05	0.94	0.94
**T2**					
Strength	1422	0.11	0.11	0.33	0.46
Cognition	1774	0.29	0.07	< 0.001	< 0.001
Adaptive Functioning (-)	1871	−0.13	0.06	0.02	0.07
Inflammation	1415	−0.33	0.19	0.08	0.21
**Epigenetic Markers**					
PhenoAge	1200	0.06	0.06	0.29	0.45
GrimAge	1199	−0.10	0.06	0.10	0.22
DunedinPACE	1200	−0.08	0.07	0.24	0.45

Values are sample size for the predictor values (N^*^; note that the N for the outcome variable is 11875 in all cases), R^2^, standardized slope (β), standard error of the slope, p-value, and q-value.

#### Within Pairs.

Results of the within-pair analyses are given in Tables S17-S19 in S1 File. Among the aging predictors, cognition and adaptive functioning significantly predicted survival at T2 but not at T1 or on average, although these did not survive multiple correction. Mean inflammation across the time points significantly predicted survival, although again this did not survive multiple correction. Among the epigenetic markers, GrimAge was the only marker significantly predicting survival within pairs.

When strength, cognition, inflammation, and adaptive functioning were included in a predictive model together with the decline epigenetic markers and PhenoAge, cognition, adaptive functioning, and inflammation significantly predicted survival at T2, and strength was significantly predictive of survival at T1. None of the epigenetic aging markers were predictive of survival within pairs.

## Discussion

Nine empirical dimensions of aging were identified here: frailty, cognition, adiposity, blood glucose, blood pressure, inflammation, blood lipids, adaptive functioning, and neurological functioning. Although these dimensions are not exhaustive of phenomena influencing or impacted by the aging process, nor of factors that might be identified from other phenomena not included here, they represent major features of aging as currently represented in commonly used and recommended aging markers. These results provide targets for future aging process research, and a common framework for conceptualizing aging processes.

Among the aging factors, cognition was the most strongly and consistently predictive of mortality, with adaptive functioning and inflammation also being strongly predictive, and glucose and strength being predictive but less consistently. Among epigenetic markers, the two later-generation, decline-predictive markers, GrimAge and DunedinPace, were most predictive of mortality, especially GrimAge. Patterns of association among the aging factors and epigenetic markers were also generally internally consistent in their associations with each other, in that the aging factors most predictive of mortality were also most strongly associated with the epigenetic markers most strongly associated with mortality.

Results point to the importance of cognition in measurement of aging, along with adaptive functioning and inflammation. In simultaneous predictive models, the aging factors, especially cognition, remained significant in predicting mortality, but the epigenetic factors diminished in predictive importance. One interpretation is that the epigenetic markers are partially redundant with the cognitive measures for predicting aging and mortality risk, that they reflect aging and mortality risk less informatively, or that they represent a subset of processes comprised by the latter. Previous analyses of data from this study [21] have demonstrated the utility of cognition in predicting mortality. Our analyses extend those results by providing information on their associations with epigenetic markers, and showing that in comparative predictive models, cognition significantly outperforms other aging indices in predicting mortality. Our results also suggest that this predictive association cannot entirely be attributed to factors operating within families of origin.

The finding that variables such as inflammation or strength did not significantly predict survival consistently after adjusting for variables such as cognition and adaptive functioning may be unexpected, given previous evidence linking them to mortality risk in older adults [22,23]. One possibility is that variation in these factors becomes more important to prediction in later life, when cumulative health burdens intensify in relevance. It is also possible that the predictive information provided by strength measures is also captured by cognition or adaptive functioning indicators more comprehensively or in a way that is functionally more commensurate with mortality risk. Future studies might explore whether the predictive validity of such biomarkers emerges more consistently in older samples or with repeated measures extending into late adulthood.

Analyses of effects within twin pairs suggest that the predictive power of the aging markers and epigenetic markers does not entirely reflect early developmental processes, at least as they occur in families of origin, as many of the effects replicated within twin pairs. At the same time, results point to the possibility that some aging measures might differ in the extent to which their associations with mortality reflect early versus later life effects. DunedinPACE and GrimAge, for example, both predicted mortality in the overall sample, but the former was less consistently associated with mortality within twin pairs, suggesting some of its association with mortality might reflect family-of-origin effects, including genetic or environmental effects shared by siblings who were raised in the same home.

An important direction for future research involves the delineation of specific causal pathways between antecedent factors, adult aging processes, and mortality. Previous research in MIDUS, for example, has illustrated the importance of stress and allostatic load in predicting aging and mortality [24–26]. These pathways likely include lifestyle behaviors such as substance use as well [26]. More careful delineation of the causal sequencing of environmental, genetic, and epigenetic variables, and possible dynamic systems pathways of behavior and health processes, is needed to better understand when and how to intervene to improve healthy aging and prevent mortality.

The current study was limited in the number of time points that some of the variables were observed, which precluded more detailed longitudinal modeling of relationships between them. For instance, observing the epigenetic variables at multiple time points in parallel with the aging markers might have afforded modeling of lagged relationships between them. Similarly, observation of the aging measures at more than two times would help characterize how change patterns in these variables relate to outcomes over time. Although the MIDUS study is reasonably representative of the US population, replication in other samples is needed, to verify the findings presented herein but also to generalize conclusions to other populations. Finally, larger samples are needed to better characterize the sources of within-family variance that might be responsible for the patterns of observed results, and to better disentangle longitudinal from cohort and other effects.

Overall, however, results presented here point to the importance of cognition among markers of aging and predictors of mortality, along with adaptive functioning and inflammation. These markers reliably predict mortality, especially cognition, and are themselves predicted by the two epigenetic markers that predicted mortality in our models.

## Supporting information

S1 File Supplement. (PDF)
