# Supplementary material for: Aging dimensions and markers as relative predictors of mortality in a longitudinal epidemiological sample
Source: PLoS One. 2025 Jun 18;20(6):e0324156. doi: 10.1371/journal.pone.0324156 (PMC12176132; doi:10.1371/journal.pone.0324156)
Supplement: S1 File — (PDF) [file pone.0324156.s001.pdf]

Supplementary Materials

Measures

Lean mass was quantified as percent lean mass using Lunar iDXA, and bone mineral density (BMD) as the 1/3 radius BMD (g/cm^2). Fasting blood lab values of hemoglobin A1C (HbA1C; %), total cholesterol (mg/dL), triglycerides (mg/dL), glucose (mg/dL), insulin (μIU/mL), IL-6 (pg/mL), IL-8 (pg/mL), IL-10 (pg/mL), creatinine (mg/dL), TNF-α (pg/mL), CRP (μg/mL), DHEA-S (μg/dL), and IGF (ng/mL) were also used in analyses. Waist-hip ratio was measured by clinical staff using a Gulik Tape Measure and computed by dividing waist measurement by the maximum hip extension measurement (both in cm). Systolic and diastolic blood pressure was taken as the mean of the second and third systolic and diastolic blood pressure measurements, respectively. Grip strength was measured via dynamometer, and timed walk and chair stand tests produced measurements of the distance walked in 6 minutes around a fixed course, and the length of time it took participants to repeatedly stand from a sitting position in a chair 5 times[1]. Visual acuity was assessed using a standard Snellen chart and averaged across left and right eyes, using the method of Holladay[2]. Peak expiratory flow (in L/min) was taken as the maximum of 3 trials using a peak flow meter. Body mass index (BMI) was computed by dividing weight (in kg) by height squared (in m) from clinical measurements. Balance was assessed using the Romberg balance task, using sway area and length measurements as two separate variables in analyses. Brain-predicted age was computed from structural brain imaging data using published algorithms.

Cognition was assessed using the Brief Test of Adult Cognition by Telephone (BTACT), a verbal measure of cognitive functioning. BTACT subtest scores used in analyses included: immediate and delayed word list learning (WL Immediate and WL Delayed), Digits Backward (DB), Category Fluency (CF), Number Sequencing (NS), and Backwards Counting (BC). Activities of daily living were measured via a self-report rating scale of difficulties performing various activities of daily living (ADL), and self-reported health was assessed via a rating scale assessing self-perceived health relative to other people. Further details regarding the measures are available at the MIDUS Colectica website (https://midus.colectica.org) or the MIDUS github site (https://midus-study.github.io).

1. Harada ND, Chiu V, Stewart, A. L. Mobility-related function in older adults: Assessment with a 6-minute walk test. Archives of Physical Medicine and Rehabilitation. 1999; 80:837-841.

2. Holladay JT. Proper method for calculating average visual acuity. Journal of Refractive Surgery. 1997; 13(4):388-391.

Table S1. Characteristics of Sample[a]

	Overall	Main Cohort	Refresher
**Age**			
Time 1	57.32 (11.55)	-	-
Time 2	58.87 (13.54)	65.96 (9.64)	52.72 (13.44)
**Gender (Female)**	52.46	52.44	52.51
**Race**			
White	78.81	82.34	72.54
Black / African American	14.90	13.11	18.1
Native American	1.21	0.79	1.97
Asian / Asian American	1.00	0.83	1.23
Other	4.08	2.91	6.15
**Education (Years)**	14.21 (2.65)	14.63 (2.58)	13.99 (2.67)
**Household Income (USD)**	73082.82 (60595.47)	77268.35 (66309.48)	71304.26 (57910.74)
**Mortality (as of 2022)**	23.41	31.12	7.71

[a]Values in columns are mean and standard deviation in parentheses, or percent.

Table S2. 6-Factor EFA Structure, First Wave[a]

	Frailty	Cognition	Adaptive Function	Adiposity	Glucose	Blood Pressure
Lean Mass	0.17	0.03	0.04	**0.97**	0.07	0.03
Bone Mineral Density	**0.73**	-0.04	-0.04	-0.06	-0.04	-0.02
Hemoglobin A1c	-0.07	-0.01	0.05	0.03	**0.82**	-0.01
Cholesterol	-0.03	0.06	-0.14	-0.15	0.11	0.10
Triglycerides	0.21	0.01	-0.01	-0.15	**0.31**	0.02
Creatinine	0.20	-0.08	0.20	0.11	-0.11	0.02
Fasting Glucose	-0.01	-0.01	-0.07	0.02	**0.95**	0.00
Fasting Insulin	0.21	0.01	0.14	-0.24	0.18	0.00
IL-6	0.03	0.02	-0.04	-0.27	0.01	0.00
IL-8	-0.10	0.04	0.27	0.26	-0.01	0.00
IL-10	-0.02	0.01	0.14	0.13	-0.01	0.03
TNF-α	0.06	-0.12	0.20	-0.08	-0.03	0.02
CRP	0.06	0.02	0.16	**-0.34**	0.06	0.05
Waist-Hip Ratio	**0.50**	-0.16	0.18	0.05	0.15	0.08
Systolic BP	-0.06	-0.01	0.01	0.01	-0.01	**1.00**
Diastolic BP	0.19	0.02	-0.11	0.02	-0.01	**0.61**
Grip Strength	**0.73**	-0.06	-0.17	0.20	-0.06	0.02
Peak Flow	**0.65**	-0.01	-0.11	0.24	-0.02	-0.04
Timed Walk	-0.05	-0.02	**0.71**	0.09	0.01	-0.01
Chair stand	-0.13	-0.02	**0.62**	0.07	-0.03	-0.01
Visual Acuity	-0.14	-0.09	0.01	-0.02	0.00	0.12
BTACT WL Immediate	-0.03	**0.90**	0.02	0.02	-0.03	0.00
BTACT WL Delayed	-0.04	**0.86**	0.00	-0.02	0.02	0.00
BTACT DB	0.04	**0.40**	-0.07	0.10	0.00	-0.01
BTACT CF	0.13	**0.31**	-0.21	0.03	-0.01	-0.03
BTACT NS	0.18	**0.30**	-0.24	0.08	-0.01	-0.04
BTACT BC	0.24	**0.30**	-0.25	0.12	0.02	-0.08
Health Rating	0.16	0.02	**0.30**	-0.14	0.03	-0.06
ADL	0.00	-0.06	**0.58**	-0.10	-0.03	-0.01
BMI	**0.48**	0.07	0.25	**-0.59**	0.13	0.08

DHEAS	**0.37**	0.08	-0.05	0.19	-0.01	-0.06
IGF	0.21	-0.04	-0.22	-0.04	-0.11	-0.05
Lean Mass	0.17	0.03	0.04	**0.97**	0.07	0.03
Bone Mineral Density	**0.73**	-0.04	-0.04	-0.06	-0.04	-0.02

[a] Values in table are standardized oblimin-rotated loadings from an exploratory factor analysis using maximum likelihood estimation. Loadings greater than or equal to 0.3 in absolute value are shown in bold.

Table S3. 6-Factor EFA Structure, Second Wave[a]

	Frailty	Cognition	Adiposity	Glucose	Blood Pressure	Neurological Function
Lean Mass	**0.31**	-0.08	**-0.75**	0.05	0.05	-0.02
Bone Mineral Density	**0.65**	-0.16	0.25	0.06	-0.05	-0.15
Hemoglobin A1c	-0.03	0.03	-0.03	**0.93**	-0.01	-0.03
Cholesterol	-0.13	0.14	-0.09	-0.02	0.08	-0.11
Triglycerides	0.20	0.02	0.14	0.24	0.03	0.06
Creatinine	0.29	-0.10	0.00	0.00	0.04	0.23
Fasting Glucose	0.04	0.03	-0.01	**0.75**	0.01	0.02
Fasting Insulin	0.10	-0.04	0.24	0.28	0.04	-0.05
IL-6	-0.05	-0.08	0.17	0.07	0.02	0.14
IL-8	-0.14	-0.07	-0.01	0.07	0.02	0.12
IL-10	-0.02	-0.02	-0.03	0.01	0.00	0.02
TNF-α	0.03	-0.04	0.10	0.00	0.03	0.16
CRP	-0.11	0.00	**0.35**	0.08	0.02	0.04
Waist-Hip Ratio	**0.59**	-0.20	0.08	0.20	0.00	0.16
Systolic BP	-0.03	0.00	0.00	0.00	**0.99**	0.03
Diastolic BP	0.10	0.04	0.04	-0.02	**0.65**	-0.13
Grip Strength	**0.79**	0.02	-0.09	-0.04	0.05	0.04
Peak Flow	**0.67**	0.07	-0.08	-0.05	0.03	-0.11
Timed Walk	-0.25	-0.20	0.27	0.10	0.02	0.11
Chair stand	-0.15	-0.15	0.19	0.03	0.08	0.14
Visual Acuity	-0.03	-0.08	-0.11	0.00	0.04	0.21
BTACT WL Immediate	-0.04	**0.89**	0.05	0.04	0.01	0.00
BTACT WL Delayed	-0.08	**0.84**	0.04	0.02	0.00	0.02
BTACT DB	0.14	**0.44**	-0.07	-0.01	-0.04	0.08
BTACT CF	0.20	**0.39**	-0.02	-0.04	-0.03	-0.11
BTACT NS	**0.34**	**0.40**	-0.11	-0.09	-0.06	0.03
BTACT BC	**0.35**	**0.41**	-0.03	-0.10	-0.04	-0.03
Brain Age	-0.08	-0.14	-0.07	0.09	0.19	**0.38**
Hearing	-0.15	-0.08	0.09	0.06	-0.06	**0.48**
Balance Area	0.02	0.00	0.04	0.00	-0.05	**0.60**

Balance Length	0.03	0.04	-0.02	-0.03	0.01	**0.94**
Health Rating	-0.04	-0.09	**0.32**	0.10	-0.01	0.02

[a] Values in table are standardized oblimin-rotated loadings from an exploratory factor analysis using maximum likelihood estimation. Loadings greater than or equal to 0.3 in absolute value are shown in bold.

Table S4. 7-Factor EFA Structure, First Wave[a]

	Frailty	Cognition: Memory	Cognition: EF	Adaptive Function	Adiposity	Glucose	Blood Pressure
Lean Mass	0.21	0.00	0.10	0.07	**-0.93**	0.07	0.04
Bone Mineral Density	**0.71**	-0.04	0.06	0.01	0.11	-0.04	-0.02
Hemoglobin A1c	-0.07	0.00	-0.05	0.04	-0.03	**0.82**	-0.01
Cholesterol	0.02	0.11	-0.08	-0.16	0.13	0.12	0.09
Triglycerides	0.21	0.04	-0.03	0.00	0.17	**0.31**	0.01
Creatinine	0.19	-0.06	-0.05	0.19	-0.10	-0.11	0.02
Fasting Glucose	-0.01	-0.01	0.02	-0.06	-0.02	**0.95**	0.00
Fasting Insulin	0.12	-0.05	0.10	0.18	0.26	0.18	0.00
IL-6	0.02	0.02	0.00	-0.03	0.27	0.00	0.00
IL-8	-0.16	-0.02	0.07	0.29	-0.25	-0.02	0.01
IL-10	-0.10	-0.08	0.15	0.19	-0.12	-0.02	0.04
TNF-α	-0.06	-0.21	0.14	0.25	0.10	-0.04	0.03
CRP	-0.03	-0.04	0.08	0.19	**0.35**	0.06	0.05
Waist-Hip Ratio	**0.49**	-0.14	-0.03	0.19	0.00	0.15	0.08
Systolic BP	-0.07	-0.02	0.00	0.01	-0.01	-0.01	**1.00**
Diastolic BP	0.24	0.06	-0.06	-0.12	-0.01	0.00	**0.60**
Grip Strength	**0.80**	-0.03	0.02	-0.13	-0.15	-0.05	0.01
Peak Flow	**0.69**	0.01	0.02	-0.07	-0.19	-0.01	-0.04
Timed Walk	-0.11	-0.01	-0.15	**0.62**	-0.08	0.02	-0.01
Chair stand	-0.14	0.02	-0.21	**0.51**	-0.07	-0.02	-0.02
Visual Acuity	-0.09	-0.04	-0.13	-0.04	0.00	0.00	0.11
BTACT WL Immediate	-0.01	**0.90**	0.02	0.02	-0.01	-0.03	0.00
BTACT WL Delayed	0.00	**0.87**	-0.01	-0.01	0.02	0.03	-0.01
BTACT DB	-0.07	0.25	**0.33**	0.03	-0.07	-0.01	0.01
BTACT CF	-0.05	0.09	**0.52**	-0.04	0.02	-0.02	-0.01
BTACT NS	-0.04	0.02	**0.65**	-0.05	-0.02	-0.04	0.00
BTACT BC	0.01	0.01	**0.69**	-0.05	-0.05	0.00	-0.04
Health Rating	0.08	-0.03	0.04	**0.30**	0.16	0.03	-0.05
ADL	-0.08	-0.07	-0.09	**0.53**	0.10	-0.03	0.00
BMI	**0.36**	0.02	0.07	**0.31**	**0.63**	0.12	0.08

DHEAS	**0.37**	0.06	0.09	-0.01	-0.16	-0.01	-0.06
IGF	0.23	-0.05	0.06	-0.19	0.05	-0.11	-0.05

[a] Values in table are standardized oblimin-rotated loadings from an exploratory factor analysis using maximum likelihood estimation. Loadings greater than or equal to 0.3 in absolute value are shown in bold.

Table S5. 7-Factor EFA Structure, Second Wave[a]

	Frailty	Cognition	Adaptive Function	Adiposity	Glucose	Blood Pressure	Neurological Function
Lean Mass	0.16	0.02	0.05	**0.98**	0.00	0.02	-0.04
Bone Mineral Density	**0.71**	-0.11	0.08	-0.07	0.04	-0.04	-0.15
Hemoglobin A1c	-0.02	0.03	0.03	0.05	**0.90**	-0.01	-0.03
Cholesterol	-0.18	0.05	-0.25	-0.09	0.01	0.08	-0.07
Triglycerides	0.24	0.02	0.02	-0.09	0.25	0.03	0.06
Creatinine	0.29	-0.04	0.15	0.14	-0.03	0.04	0.18
Fasting Glucose	0.01	-0.02	-0.13	-0.06	**0.79**	0.01	0.03
Fasting Insulin	0.19	0.02	0.20	-0.12	0.25	0.03	-0.06
IL-6	0.04	0.00	0.27	-0.06	0.03	0.02	0.11
IL-8	-0.11	0.02	0.24	0.14	0.05	0.01	0.08
IL-10	0.00	0.04	0.16	0.14	-0.01	-0.01	0.01
TNF-α	0.08	0.02	0.19	0.00	-0.02	0.03	0.14
CRP	0.02	0.03	0.18	**-0.32**	0.07	0.03	0.02
Waist-Hip Ratio	**0.62**	-0.13	0.16	0.12	0.17	0.00	0.13
Systolic BP	-0.03	0.01	0.05	0.04	0.00	**0.99**	0.03
Diastolic BP	0.08	-0.02	-0.15	-0.12	0.00	**0.65**	-0.11
Grip Strength	**0.69**	-0.02	-0.24	0.14	-0.04	0.06	0.06
Peak Flow	**0.60**	0.04	-0.20	0.11	-0.07	0.03	-0.10
Timed Walk	-0.08	-0.02	**0.60**	-0.04	0.03	0.00	0.04
Chair stand	-0.01	0.01	**0.50**	-0.02	-0.05	0.07	0.07
Visual Acuity	-0.07	-0.11	-0.08	0.03	0.02	0.05	0.19
BTACT WL Immediate	-0.02	**0.91**	0.02	-0.01	0.01	0.01	-0.01
BTACT WL Delayed	-0.06	**0.86**	0.03	0.01	0.00	-0.01	0.01
BTACT DB	0.08	**0.39**	-0.14	0.09	0.00	-0.04	0.09
BTACT CF	0.14	**0.30**	-0.28	-0.06	-0.02	-0.02	-0.07
BTACT NS	0.24	0.29	**-0.33**	0.05	-0.06	-0.05	0.06
BTACT BC	0.27	**0.30**	**-0.33**	-0.05	-0.07	-0.03	-0.01
Brain Age	-0.10	-0.14	0.05	0.01	0.09	0.19	**0.33**

Hearing	-0.11	-0.02	0.24	-0.02	0.05	-0.06	**0.42**
Balance Area	0.03	0.01	0.12	0.02	0.00	-0.04	**0.57**
Balance Length	0.01	0.00	-0.04	-0.03	-0.01	0.01	**1.00**
Health Rating	0.10	0.01	**0.35**	-0.17	0.06	-0.02	-0.01
ADL	-0.02	-0.08	**0.56**	-0.11	0.00	0.03	0.02
BMI	**0.36**	0.05	0.24	**-0.68**	0.05	0.06	-0.03
DHEAS	0.29	0.02	-0.19	0.09	-0.06	-0.03	-0.14
IGF	0.21	0.03	-0.21	0.09	-0.06	-0.10	-0.08

[a] Values in table are standardized oblimin-rotated loadings from an exploratory factor analysis using maximum likelihood estimation. Loadings greater than or equal to 0.3 in absolute value are shown in bold.

Table S6. 8-Factor EFA Structure, First Wave[a]

	Frailty	Cognition: Memory	Cognition: EF	Adaptive Function	Adiposity	Glucose	Blood Pressure	Inflammation
Lean Mass	0.23	0.00	0.11	0.06	**-0.91**	0.08	0.04	0.08
Bone Mineral Density	**0.71**	-0.04	0.06	-0.01	0.12	-0.04	-0.02	-0.02
Hemoglobin A1c	-0.07	0.01	-0.05	0.04	-0.03	**0.82**	-0.01	0.00
Cholesterol	0.00	0.11	-0.08	-0.13	0.11	0.11	0.10	-0.07
Triglycerides	0.21	0.04	-0.04	-0.02	0.17	**0.31**	0.01	0.00
Creatinine	0.21	-0.05	-0.05	0.15	-0.07	-0.11	0.02	0.08
Fasting Glucose	-0.01	-0.01	0.02	-0.05	-0.01	**0.94**	0.00	-0.01
Fasting Insulin	0.13	-0.04	0.09	0.13	0.29	0.18	0.00	0.06
IL-6	0.02	0.02	-0.03	-0.08	0.29	0.00	-0.01	0.07
IL-8	-0.06	0.05	-0.07	-0.03	-0.10	-0.02	-0.02	**0.64**
IL-10	-0.02	-0.03	0.02	-0.10	0.01	-0.02	0.01	**0.53**
TNF-α	0.00	-0.17	0.06	0.06	0.19	-0.04	0.02	**0.33**
CRP	-0.01	-0.03	0.05	0.10	**0.39**	0.06	0.04	0.15
Waist-Hip Ratio	**0.50**	-0.14	-0.03	0.16	0.02	0.15	0.08	0.03
Systolic BP	-0.07	-0.02	0.00	0.00	-0.01	-0.01	**1.00**	0.02
Diastolic BP	0.22	0.05	-0.03	-0.06	-0.03	0.00	**0.60**	-0.10
Grip Strength	**0.79**	-0.03	0.01	-0.14	-0.14	-0.05	0.01	-0.03
Peak Flow	**0.69**	0.01	0.02	-0.08	-0.19	-0.01	-0.04	-0.03
Timed Walk	-0.09	-0.02	-0.06	**0.71**	-0.09	0.00	-0.01	-0.02
Chair stand	-0.12	0.01	-0.15	**0.56**	-0.07	-0.03	-0.02	0.01
Visual Acuity	-0.09	-0.04	-0.14	-0.03	-0.01	0.00	0.11	-0.01
BTACT WL Immediate	0.00	**0.92**	0.01	0.01	-0.01	-0.03	0.00	0.02
BTACT WL Delayed	-0.01	**0.85**	0.00	-0.01	0.02	0.03	-0.01	-0.02
BTACT DB	-0.07	0.24	**0.34**	0.04	-0.07	-0.01	0.01	-0.02
BTACT CF	-0.06	0.09	**0.52**	-0.04	0.02	-0.02	0.00	-0.01
BTACT NS	-0.04	0.02	**0.64**	-0.06	-0.01	-0.04	0.00	0.02
BTACT BC	-0.02	-0.01	**0.74**	-0.02	-0.05	0.00	-0.03	-0.04
Health Rating	0.08	-0.03	0.08	**0.33**	0.16	0.02	-0.05	-0.03
ADL	-0.06	-0.07	-0.04	**0.55**	0.11	-0.03	0.00	0.01
BMI	**0.37**	0.03	0.06	0.24	**0.66**	0.12	0.08	0.06

DHEAS	**0.38**	0.06	0.08	-0.03	-0.15	-0.01	-0.06	0.03
IGF	0.21	-0.06	0.07	-0.15	0.04	-0.11	-0.05	-0.07

[a] Values in table are standardized oblimin-rotated loadings from an exploratory factor analysis using maximum likelihood estimation. Loadings greater than or equal to 0.3 in absolute value are shown in bold.

Table S7. 8-Factor EFA Structure, Second Wave[a]

	Frailty	Cognition: Memory	Cognition: EF	Adaptive Function	Adiposity	Glucose	Blood Pressure	Neurological Function
Lean Mass	0.14	0.00	0.02	0.05	**0.98**	0.00	0.03	-0.04
Bone Mineral Density	**0.73**	-0.06	-0.05	0.09	-0.08	0.03	-0.05	-0.14
Hemoglobin A1c	-0.03	0.03	-0.03	0.03	0.05	**0.90**	-0.01	-0.03
Cholesterol	-0.17	0.03	0.09	-0.20	-0.09	0.01	0.08	-0.07
Triglycerides	0.17	-0.03	0.12	0.10	-0.09	0.25	0.04	0.06
Creatinine	0.29	-0.02	-0.06	0.13	0.14	-0.03	0.03	0.19
Fasting Glucose	0.01	-0.02	0.01	-0.11	-0.06	**0.79**	0.01	0.03
Fasting Insulin	0.13	-0.02	0.05	0.24	-0.11	0.26	0.04	-0.07
IL-6	0.01	0.00	-0.05	0.26	-0.05	0.03	0.02	0.11
IL-8	-0.10	0.04	-0.12	0.18	0.15	0.04	0.01	0.08
IL-10	-0.04	0.01	0.02	0.17	0.15	-0.01	0.00	0.00
TNF-α	0.05	0.01	-0.03	0.18	0.00	-0.02	0.03	0.13
CRP	-0.02	0.01	0.00	0.19	**-0.31**	0.07	0.03	0.02
Waist-Hip Ratio	**0.56**	-0.15	0.06	0.21	0.12	0.17	0.00	0.13
Systolic BP	-0.04	0.00	-0.01	0.04	0.04	0.00	**0.99**	0.03
Diastolic BP	0.11	0.00	0.01	-0.14	-0.13	0.00	**0.65**	-0.10
Grip Strength	**0.74**	0.01	0.05	-0.21	0.12	-0.04	0.05	0.08
Peak Flow	**0.61**	0.05	0.10	-0.15	0.09	-0.06	0.03	-0.09
Timed Walk	-0.15	-0.03	-0.12	**0.55**	-0.02	0.02	0.01	0.03
Chair stand	-0.05	0.01	-0.12	**0.45**	0.00	-0.05	0.07	0.06
Visual Acuity	-0.06	-0.10	0.00	-0.08	0.03	0.02	0.05	0.19
BTACT WL Immediate	0.01	**0.91**	0.03	0.01	-0.01	0.01	0.01	-0.01
BTACT WL Delayed	-0.01	**0.86**	-0.02	-0.01	0.01	0.00	-0.01	0.01
BTACT DB	-0.10	0.19	**0.46**	0.08	0.10	0.03	-0.02	0.08
BTACT CF	-0.05	0.08	**0.54**	-0.03	-0.05	0.01	0.00	-0.08
BTACT NS	0.01	0.02	**0.66**	-0.03	0.06	-0.02	-0.03	0.05
BTACT BC	0.01	-0.01	**0.76**	0.01	-0.04	-0.03	0.01	-0.01
Brain Age	-0.04	-0.07	-0.17	-0.05	0.00	0.08	0.19	**0.33**

Hearing	-0.04	0.07	-0.25	0.11	-0.03	0.03	-0.07	**0.42**
Balance Area	0.00	-0.01	0.01	0.13	0.02	0.00	-0.04	**0.57**
Balance Length	0.01	0.00	0.02	-0.03	-0.03	-0.01	0.01	**1.00**
Health Rating	0.00	-0.05	0.06	**0.41**	-0.15	0.06	-0.01	-0.02
ADL	-0.09	-0.10	-0.09	**0.54**	-0.09	-0.01	0.03	0.01
BMI	**0.32**	0.04	0.00	0.26	**-0.68**	0.05	0.06	-0.03
DHEAS	0.25	-0.02	0.16	-0.10	0.09	-0.05	-0.03	-0.14
IGF	0.23	0.03	0.07	-0.17	0.08	-0.06	-0.10	-0.08

[a] Values in table are standardized oblimin-rotated loadings from an exploratory factor analysis using maximum likelihood estimation. Loadings greater than or equal to 0.3 in absolute value are shown in bold.

Table S8. 9-Factor EFA Structure, First Wave[a]

	Frailty	Cognition: Memory	Cognition: EF	Adaptive Function	Adiposity	Glucose	Blood Pressure	Lipids	Inflammation
Lean Mass	0.25	0.00	0.11	0.06	**-0.89**	0.08	0.04	-0.03	0.08
Bone Mineral Density	**0.71**	-0.04	0.05	-0.01	0.13	-0.04	-0.02	0.03	-0.02
Hemoglobin A1c	-0.05	0.01	-0.05	0.00	-0.01	**0.89**	-0.01	-0.04	-0.01
Cholesterol	-0.09	0.08	-0.05	-0.11	0.03	-0.01	0.09	**0.36**	-0.08
Triglycerides	0.00	0.00	0.00	0.00	-0.01	0.01	-0.01	**1.00**	0.01
Creatinine	0.21	-0.05	-0.05	0.14	-0.07	-0.11	0.02	0.01	0.08
Fasting Glucose	-0.01	-0.02	0.03	-0.04	-0.02	**0.87**	0.00	0.06	-0.01
Fasting Insulin	0.11	-0.05	0.10	0.12	0.28	0.16	0.00	0.09	0.06
IL-6	0.03	0.03	-0.03	-0.08	**0.30**	0.01	-0.01	-0.03	0.07
IL-8	-0.07	0.05	-0.07	-0.02	-0.09	-0.03	-0.02	0.02	**0.64**
IL-10	-0.02	-0.02	0.02	-0.10	0.02	-0.02	0.01	-0.02	**0.54**
TNF-α	-0.01	-0.17	0.06	0.06	0.19	-0.05	0.02	0.02	**0.33**
CRP	0.00	-0.02	0.04	0.09	**0.41**	0.08	0.04	-0.05	0.14
Waist-Hip Ratio	**0.48**	-0.15	-0.02	0.15	0.01	0.12	0.08	0.10	0.03
Systolic BP	-0.07	-0.02	0.00	0.00	0.00	-0.01	**1.00**	-0.01	0.02
Diastolic BP	0.21	0.05	-0.02	-0.06	-0.03	-0.01	**0.60**	0.02	-0.11
Grip Strength	**0.79**	-0.03	0.01	-0.14	-0.14	-0.06	0.01	0.03	-0.03
Peak Flow	**0.70**	0.01	0.02	-0.08	-0.17	0.00	-0.04	-0.01	-0.03
Timed Walk	-0.09	-0.02	-0.06	**0.71**	-0.08	0.01	0.00	-0.03	-0.02
Chair stand	-0.13	0.01	-0.15	**0.56**	-0.07	-0.04	-0.02	0.03	0.01
Visual Acuity	-0.10	-0.04	-0.13	-0.03	-0.01	0.00	0.11	0.00	-0.01
BTACT WL Immediate	0.00	**0.91**	0.01	0.01	0.00	-0.02	0.00	0.01	0.02
BTACT WL Delayed	0.00	**0.85**	0.00	-0.01	0.02	0.02	-0.01	0.00	-0.02
BTACT DB	-0.07	0.24	**0.34**	0.04	-0.07	-0.01	0.01	-0.01	-0.02
BTACT CF	-0.07	0.09	**0.52**	-0.05	0.01	-0.03	0.00	0.04	-0.01
BTACT NS	-0.05	0.02	**0.64**	-0.06	-0.02	-0.05	0.00	0.02	0.02
BTACT BC	-0.01	-0.01	**0.74**	-0.02	-0.04	0.00	-0.03	-0.03	-0.04
Health Rating	0.05	-0.04	0.09	**0.34**	0.14	-0.02	-0.05	0.13	-0.04
ADL	-0.08	-0.08	-0.04	**0.55**	0.11	-0.04	0.00	0.04	0.01
BMI	**0.37**	0.04	0.05	0.23	**0.67**	0.13	0.08	0.02	0.06

DHEAS	**0.38**	0.06	0.09	-0.03	-0.14	-0.01	-0.05	0.00	0.02
IGF	0.23	-0.05	0.06	-0.15	0.05	-0.09	-0.05	-0.09	-0.07

[a] Values in table are standardized oblimin-rotated loadings from an exploratory factor analysis using maximum likelihood estimation. Loadings greater than or equal to 0.3 in absolute value are shown in bold.

Table S9. 9-Factor EFA Structure, Second Wave[a]

	Frailty	Cognition: Memory	Cognition: EF	Adaptive Function	Adiposity	Glucose	Blood Pressure	Brain Age	Vestibular Function
Lean Mass	0.14	0.00	0.01	0.05	**0.98**	0.01	-0.01	-0.03	-0.03
Bone Mineral Density	**0.73**	-0.06	-0.06	0.06	-0.08	0.04	-0.03	-0.08	-0.12
Hemoglobin A1c	-0.03	0.03	-0.03	0.03	0.05	**0.90**	-0.02	0.02	-0.04
Cholesterol	-0.19	0.03	0.10	-0.18	-0.07	0.01	0.13	0.04	-0.08
Triglycerides	0.16	-0.03	0.14	0.12	-0.08	0.25	0.05	0.03	0.05
Creatinine	**0.30**	-0.01	-0.03	0.13	0.13	-0.04	-0.01	0.14	0.14
Fasting Glucose	0.01	-0.03	0.01	-0.11	-0.05	**0.79**	0.03	-0.02	0.05
Fasting Insulin	0.13	-0.02	0.05	0.25	-0.11	0.26	0.00	0.00	-0.06
IL-6	0.00	0.00	-0.04	0.26	-0.04	0.03	0.02	0.02	0.10
IL-8	-0.11	0.04	-0.08	0.20	0.15	0.03	0.03	0.13	0.04
IL-10	-0.05	0.01	0.02	0.18	0.16	-0.01	0.04	-0.02	0.01
TNF-α	0.05	0.01	-0.06	0.19	0.02	-0.01	0.02	-0.12	0.18
CRP	-0.03	0.00	-0.01	0.20	**-0.30**	0.08	0.05	-0.06	0.05
Waist-Hip Ratio	**0.55**	-0.15	0.09	0.21	0.12	0.16	0.03	0.09	0.10
Systolic BP	-0.02	0.02	-0.04	0.14	0.11	0.03	**0.64**	0.13	0.07
Diastolic BP	0.01	0.00	0.00	-0.03	-0.04	-0.01	**1.00**	-0.03	-0.03
Grip Strength	**0.72**	0.01	0.07	-0.20	0.13	-0.04	0.09	0.02	0.07
Peak Flow	**0.61**	0.05	0.11	-0.15	0.09	-0.06	0.02	0.01	-0.09
Timed Walk	-0.14	-0.04	-0.12	**0.55**	-0.02	0.02	-0.02	-0.01	0.04
Chair stand	-0.03	0.02	-0.09	**0.46**	0.00	-0.06	-0.01	0.16	0.01
Visual Acuity	-0.07	-0.10	0.02	-0.06	0.04	0.02	0.05	0.06	0.18
BTACT WL Immediate	0.02	**0.92**	0.03	0.01	-0.01	0.01	0.00	-0.01	-0.01
BTACT WL Delayed	-0.02	**0.85**	-0.02	-0.01	0.00	-0.01	0.00	0.01	0.01
BTACT DB	-0.11	0.18	**0.44**	0.08	0.10	0.03	0.02	-0.09	0.10
BTACT CF	-0.04	0.08	**0.53**	-0.03	-0.06	0.01	-0.01	-0.02	-0.09
BTACT NS	0.02	0.02	**0.69**	-0.03	0.05	-0.03	-0.04	0.05	0.02
BTACT BC	0.01	-0.01	**0.76**	0.02	-0.05	-0.03	0.00	-0.02	-0.02
Brain Age	0.01	-0.01	0.00	-0.01	-0.02	0.01	0.01	**1.00**	0.00

Hearing	-0.03	0.07	-0.21	0.10	-0.04	0.02	-0.07	0.17	**0.36**
Balance Area	-0.01	-0.01	-0.02	0.12	0.03	0.00	0.02	-0.08	**0.59**
Balance Length	0.00	-0.01	0.01	-0.03	-0.03	0.00	-0.01	0.01	**1.00**
Health Rating	-0.02	-0.06	0.03	**0.42**	-0.13	0.07	0.06	-0.15	0.03
ADL	-0.09	-0.10	-0.09	**0.55**	-0.08	-0.01	0.03	0.02	0.01
BMI	**0.34**	0.04	-0.02	0.25	**-0.68**	0.06	0.00	-0.02	-0.02
DHEAS	0.23	-0.03	0.09	-0.12	0.10	-0.03	0.08	-0.27	-0.05
IGF	0.21	0.02	0.01	-0.21	0.08	-0.04	-0.01	-0.25	0.00

[a] Values in table are standardized oblimin-rotated loadings from an exploratory factor analysis using maximum likelihood estimation. Loadings greater than or equal to 0.3 in absolute value are shown in bold.

Table S10. 10-Factor EFA Structure, First Wave[a]

	Frailty	Cognition: Memory	Cognition: EF	Adaptive Function	Adiposity	Glucose	Blood Pressure	Lipids	Inflammation	BMI
Lean Mass	0.28	-0.02	0.13	0.04	**-0.69**	0.09	0.04	-0.03	0.11	-0.25
Bone Mineral Density	**0.62**	-0.04	0.04	-0.05	0.02	-0.05	-0.03	0.04	-0.04	0.22
Hemoglobin A1c	-0.06	0.01	-0.05	0.01	-0.03	**0.85**	-0.01	-0.04	-0.01	0.05
Cholesterol	-0.06	0.08	-0.03	-0.07	0.08	0.00	0.09	**0.36**	-0.08	-0.08
Triglycerides	0.01	0.00	0.00	0.00	-0.01	0.01	-0.01	**1.00**	0.01	-0.01
Creatinine	**0.31**	-0.03	-0.02	0.22	0.09	-0.08	0.02	0.01	0.11	-0.12
Fasting Glucose	0.01	-0.01	0.03	-0.03	0.01	**0.90**	0.00	0.05	-0.01	-0.01
Fasting Insulin	0.07	-0.04	0.08	0.10	0.17	0.15	0.00	0.10	0.04	0.18
IL-6	0.15	0.08	-0.02	0.03	**0.42**	0.05	0.01	-0.03	0.09	-0.12
IL-8	-0.08	0.04	-0.07	-0.03	-0.10	-0.03	-0.02	0.02	**0.62**	0.05
IL-10	0.02	0.00	0.01	-0.07	0.08	0.00	0.02	-0.02	**0.54**	-0.02
TNF-α	0.05	-0.15	0.08	0.12	0.24	-0.02	0.02	0.03	**0.35**	0.01
CRP	0.05	0.01	0.05	0.15	**0.41**	0.10	0.05	-0.05	0.15	0.07
Waist-Hip Ratio	**0.52**	-0.12	-0.01	0.19	0.06	0.14	0.08	0.10	0.04	0.04
Systolic BP	-0.08	-0.03	-0.01	-0.01	-0.03	-0.01	**0.99**	0.00	0.02	0.05
Diastolic BP	0.29	0.06	0.00	0.01	0.08	0.01	**0.61**	0.02	-0.09	-0.11
Grip Strength	**0.77**	-0.02	0.02	-0.14	-0.12	-0.05	0.01	0.03	-0.03	0.06
Peak Flow	**0.68**	0.02	0.02	-0.08	-0.15	0.00	-0.04	-0.01	-0.02	0.04
Timed Walk	-0.04	0.00	-0.04	**0.72**	-0.05	0.02	0.00	-0.03	-0.02	0.03
Chair stand	-0.12	0.02	-0.13	**0.54**	-0.07	-0.04	-0.02	0.03	0.01	0.04
Visual Acuity	-0.11	-0.04	-0.14	-0.05	-0.02	0.00	0.11	0.00	-0.01	-0.01
BTACT WL Immediate	-0.03	**0.86**	0.04	-0.01	-0.03	-0.04	-0.01	0.01	0.02	0.04
BTACT WL Delayed	0.03	**0.90**	-0.01	0.01	0.04	0.03	0.00	0.00	0.00	-0.02
BTACT DB	-0.05	0.25	**0.34**	0.05	-0.04	-0.01	0.01	-0.01	-0.01	-0.03
BTACT CF	-0.09	0.08	**0.52**	-0.06	-0.02	-0.04	0.00	0.05	-0.01	0.05
BTACT NS	-0.04	0.03	**0.64**	-0.05	-0.01	-0.04	0.00	0.02	0.02	0.02
BTACT BC	0.00	-0.01	**0.75**	-0.01	-0.01	0.01	-0.02	-0.03	-0.03	0.01
Health Rating	0.08	-0.03	0.10	**0.34**	0.15	0.00	-0.05	0.13	-0.04	0.05

ADL	-0.09	-0.07	-0.04	**0.50**	0.06	-0.04	0.00	0.04	0.00	0.12
BMI	0.08	0.01	0.01	0.05	0.04	0.04	0.03	0.01	0.01	**0.94**
DHEAS	**0.37**	0.06	0.10	-0.02	-0.12	-0.01	-0.06	-0.01	0.03	0.03
IGF	**0.34**	-0.02	0.10	-0.04	0.22	-0.05	-0.04	-0.10	-0.05	-0.17

[a] Values in table are standardized oblimin-rotated loadings from an exploratory factor analysis using maximum likelihood estimation. Loadings greater than or equal to 0.3 in absolute value are shown in bold.

Table S11. 10-Factor EFA Structure, Second Wave[a]

	Frailty	Cognition: Memory	Cognition: EF	Adaptive Function	Adiposity	Glucose	Blood Pressure	Inflammation	Brain Age	Vestibular Function
Lean Mass	0.14	0.00	0.01	0.02	**0.98**	0.01	-0.01	0.07	-0.04	-0.03
Bone Mineral Density	**0.75**	-0.06	-0.05	0.08	-0.08	0.05	-0.03	-0.01	-0.08	-0.11
Hemoglobin A1c	-0.03	0.03	-0.03	0.02	0.05	**0.88**	-0.02	0.03	0.03	-0.04
Cholesterol	-0.16	0.04	0.11	-0.04	-0.06	0.02	0.13	-0.19	0.04	-0.07
Triglycerides	0.14	-0.04	0.13	0.02	-0.09	0.25	0.05	0.13	0.03	0.04
Creatinine	0.28	-0.02	-0.03	0.04	0.12	-0.05	-0.01	0.15	0.13	0.13
Fasting Glucose	0.01	-0.02	0.01	-0.06	-0.04	**0.81**	0.03	-0.08	-0.02	0.05
Fasting Insulin	0.09	-0.03	0.04	0.04	-0.14	0.24	-0.01	0.27	0.00	-0.08
IL-6	-0.05	-0.02	-0.06	0.04	-0.07	0.00	0.01	**0.33**	0.01	0.08
IL-8	-0.19	0.03	-0.12	-0.11	0.12	-0.01	0.02	**0.42**	0.13	0.01
IL-10	-0.08	0.00	0.02	0.04	0.14	-0.03	0.04	0.21	-0.02	-0.01
TNF-α	-0.02	-0.01	-0.07	-0.06	-0.01	-0.05	0.02	**0.35**	-0.12	0.15
CRP	-0.06	0.00	-0.02	0.03	**-0.32**	0.06	0.04	0.23	-0.06	0.03
Waist-Hip Ratio	**0.53**	-0.16	0.09	0.08	0.11	0.15	0.03	0.21	0.09	0.09
Systolic BP	-0.03	0.01	-0.04	0.09	0.10	0.02	**0.64**	0.09	0.12	0.07
Diastolic BP	0.01	0.00	0.00	-0.02	-0.04	0.00	**1.00**	-0.02	-0.03	-0.03
Grip Strength	**0.71**	0.01	0.06	-0.17	0.14	-0.04	0.09	-0.05	0.03	0.07
Peak Flow	**0.60**	0.05	0.12	-0.11	0.10	-0.06	0.02	-0.05	0.02	-0.09
Timed Walk	-0.10	-0.04	-0.05	**0.68**	0.00	0.04	-0.01	0.04	-0.05	0.03
Chair stand	0.04	0.02	-0.01	**0.70**	0.02	-0.04	0.01	-0.10	0.12	0.02
Visual Acuity	-0.07	-0.10	0.02	-0.03	0.04	0.03	0.05	-0.04	0.06	0.18
BTACT WL Immediate	0.01	**0.91**	0.03	0.01	-0.01	0.01	0.00	0.01	-0.01	-0.01
BTACT WL Delayed	-0.01	**0.86**	-0.02	0.00	0.00	0.00	0.00	0.00	0.00	0.01
BTACT DB	-0.11	0.18	**0.44**	0.03	0.10	0.03	0.02	0.05	-0.09	0.10
BTACT CF	-0.04	0.08	**0.54**	0.00	-0.05	0.02	0.00	-0.04	-0.02	-0.08
BTACT NS	0.02	0.02	**0.67**	-0.04	0.05	-0.02	-0.04	-0.01	0.05	0.03
BTACT BC	0.00	-0.02	**0.77**	0.01	-0.04	-0.03	0.00	0.01	-0.02	-0.02
Brain Age	0.01	-0.01	0.00	0.01	-0.02	0.01	0.01	0.00	**0.99**	0.01

Hearing	-0.01	0.07	-0.18	0.18	-0.03	0.03	-0.07	-0.05	0.16	**0.36**
Balance Area	0.01	-0.01	0.00	0.16	0.03	0.02	0.02	-0.03	-0.09	**0.59**
Balance Length	0.00	-0.01	0.00	-0.03	-0.03	0.00	-0.01	0.01	0.02	**0.99**
Health Rating	-0.02	-0.07	0.03	0.25	-0.16	0.07	0.06	0.22	-0.17	0.02
ADL	-0.09	-0.10	-0.09	**0.38**	-0.11	0.00	0.02	0.26	-0.01	0.00
BMI	**0.31**	0.04	-0.02	0.10	**-0.70**	0.05	0.00	0.21	-0.03	-0.03
DHEAS	0.25	-0.02	0.10	-0.05	0.11	-0.02	0.08	-0.11	-0.26	-0.04
IGF	0.24	0.03	0.02	-0.06	0.10	-0.03	0.00	-0.21	-0.24	0.01

[a] Values in table are standardized oblimin-rotated loadings from an exploratory factor analysis using maximum likelihood estimation. Loadings greater than or equal to 0.3 in absolute value are shown in bold.

Table S12. 11-Factor EFA Structure, First Wave[a]

	Frailty	Cognition: Memory	Cognition: EF	Adaptive Function	Adiposity	Glucose	Blood Pressure	Lipids	Inflammation	BMI	Perceived Health
Lean Mass	0.28	-0.02	0.13	0.06	**-0.68**	0.09	0.05	-0.03	0.12	-0.25	-0.04
Bone Mineral Density	**0.62**	-0.04	0.04	-0.07	0.01	-0.05	-0.03	0.04	-0.04	0.23	0.02
Hemoglobin A1c	-0.06	0.01	-0.05	0.01	-0.03	**0.85**	-0.01	-0.04	0.00	0.04	0.00
Cholesterol	-0.06	0.08	-0.04	-0.08	0.07	0.00	0.09	**0.35**	-0.08	-0.08	0.01
Triglycerides	0.01	0.00	0.00	0.00	-0.01	0.01	-0.01	**1.00**	0.01	-0.01	0.01
Creatinine	**0.32**	-0.04	0.01	0.27	0.12	-0.08	0.02	0.02	0.10	-0.12	-0.04
Fasting Glucose	0.01	-0.01	0.03	-0.03	0.01	**0.90**	0.00	0.05	-0.01	-0.01	0.00
Fasting Insulin	0.07	-0.04	0.08	0.08	0.17	0.15	0.00	0.09	0.04	0.19	0.04
IL-6	0.14	0.08	-0.02	0.03	**0.42**	0.05	0.01	-0.03	0.08	-0.11	0.03
IL-8	-0.09	0.05	-0.07	-0.02	-0.10	-0.02	-0.02	0.02	**0.62**	0.05	0.00
IL-10	0.01	0.00	-0.01	-0.09	0.07	0.00	0.02	-0.02	**0.55**	-0.01	0.02
TNF-α	0.06	-0.15	0.10	0.15	0.27	-0.02	0.02	0.03	**0.34**	0.01	-0.03
CRP	0.05	0.01	0.05	0.14	**0.40**	0.10	0.05	-0.05	0.14	0.08	0.04
Waist-Hip Ratio	**0.52**	-0.13	0.00	0.19	0.07	0.14	0.08	0.10	0.04	0.04	0.02
Systolic BP	-0.08	-0.02	-0.01	-0.01	-0.03	-0.01	**0.99**	-0.01	0.02	0.05	0.00
Diastolic BP	0.29	0.06	0.00	0.02	0.08	0.01	**0.61**	0.02	-0.10	-0.11	0.00
Grip Strength	**0.77**	-0.02	0.02	-0.13	-0.12	-0.05	0.01	0.03	-0.03	0.06	-0.01
Peak Flow	**0.69**	0.02	0.03	-0.06	-0.14	0.00	-0.04	-0.01	-0.03	0.03	-0.03
Timed Walk	-0.05	-0.01	-0.04	**0.70**	-0.03	0.02	-0.01	-0.03	-0.03	0.05	0.05

Chair stand	-0.10	0.01	-0.11	**0.60**	-0.04	-0.04	-0.02	0.04	-0.01	0.05	-0.05
Visual Acuity	-0.10	-0.04	-0.13	-0.03	-0.02	0.00	0.11	0.00	-0.01	-0.01	-0.04
BTACT WL Immediate	-0.03	**0.87**	0.03	-0.01	-0.03	-0.04	-0.01	0.01	0.02	0.03	0.00
BTACT WL Delayed	0.03	**0.90**	-0.01	0.02	0.04	0.03	0.00	0.00	0.00	-0.02	0.00
BTACT DB	-0.05	0.25	**0.35**	0.06	-0.03	-0.01	0.01	-0.01	-0.02	-0.03	-0.01
BTACT CF	-0.09	0.08	**0.54**	-0.03	0.00	-0.04	-0.01	0.05	-0.01	0.05	-0.04
BTACT NS	-0.04	0.03	**0.64**	-0.06	-0.01	-0.04	0.00	0.02	0.02	0.02	0.01
BTACT BC	0.00	-0.01	**0.76**	-0.01	0.00	0.01	-0.02	-0.03	-0.04	0.01	0.01
Health Rating	0.01	0.00	0.00	0.00	-0.01	0.00	0.00	0.01	0.00	-0.01	**1.00**
ADL	-0.12	-0.07	-0.09	**0.36**	0.01	-0.03	0.00	0.02	0.01	0.14	0.27
BMI	0.08	0.00	0.01	0.04	0.04	0.04	0.03	0.01	0.01	**0.95**	0.01
DHEAS	**0.36**	0.06	0.07	-0.09	-0.15	-0.01	-0.05	-0.02	0.05	0.03	0.12
IGF	**0.33**	-0.02	0.07	-0.10	0.19	-0.05	-0.03	-0.11	-0.05	-0.17	0.10

[a] Values in table are standardized oblimin-rotated loadings from an exploratory factor analysis using maximum likelihood estimation. Loadings greater than or equal to 0.3 in absolute value are shown in bold.

Table S13. 11-Factor EFA Structure, Second Wave[a]

	Frailty	Cognition: Memory	Cognition: EF	Adaptive Function	Adiposity	Glucose	Blood Pressure	Lipids	Inflammation	Vestibular Function	Brain Age
Lean Mass	0.14	0.00	0.01	0.02	**0.98**	0.01	0.03	0.00	0.08	-0.04	-0.04
Bone Mineral Density	**0.74**	-0.06	-0.06	0.09	-0.08	0.03	-0.04	0.04	0.00	-0.11	-0.07
Hemoglobin A1c	-0.02	0.01	0.00	0.01	0.03	**1.01**	0.00	-0.03	0.00	-0.02	0.01
Cholesterol	-0.17	0.06	0.04	-0.05	-0.03	-0.03	0.06	0.26	**-0.30**	-0.08	0.03
Triglycerides	0.00	0.01	0.00	0.00	0.01	0.00	0.00	**1.00**	-0.01	0.01	0.00
Creatinine	0.26	-0.02	-0.02	0.04	0.12	-0.04	0.01	0.04	0.15	0.13	0.13
Fasting Glucose	0.03	-0.03	-0.02	-0.06	-0.07	**0.67**	0.03	0.09	-0.07	0.06	-0.02
Fasting Insulin	0.06	-0.03	0.02	0.04	-0.13	0.21	0.04	0.18	0.22	-0.08	-0.02
IL-6	-0.05	-0.02	-0.05	0.03	-0.08	0.03	0.02	0.01	**0.30**	0.08	0.02
IL-8	-0.20	0.03	-0.11	-0.13	0.10	-0.01	-0.01	0.02	**0.43**	0.00	0.15
IL-10	-0.07	0.00	0.02	0.04	0.13	-0.02	0.00	-0.01	0.20	-0.01	-0.01
TNF-α	-0.04	0.00	-0.07	-0.05	-0.01	-0.03	0.05	0.08	**0.30**	0.15	-0.12
CRP	-0.06	0.00	-0.02	0.02	**-0.33**	0.07	0.04	0.02	0.20	0.03	-0.06
Waist-Hip Ratio	**0.51**	-0.15	0.07	0.07	0.10	0.12	-0.02	0.15	0.21	0.09	0.10
Systolic BP	-0.04	**-0.01**	-0.01	0.04	0.04	0.01	**0.99**	-0.01	0.02	0.03	0.01
Diastolic BP	0.11	**0.00**	-0.01	-0.11	-0.12	-0.01	**0.65**	0.02	-0.08	-0.09	-0.01
Grip Strength	**0.73**	0.01	0.07	-0.17	0.12	-0.03	0.05	-0.01	-0.04	0.07	0.04
Peak Flow	**0.59**	0.04	0.13	-0.10	0.10	-0.04	0.03	0.01	-0.05	-0.09	0.01
Timed Walk	-0.10	-0.04	-0.05	**0.66**	-0.01	0.05	0.01	-0.02	0.05	0.03	-0.04

Chair stand	0.03	0.03	-0.01	**0.70**	0.03	-0.03	0.04	0.02	-0.10	0.01	0.11
Visual Acuity	-0.06	-0.11	0.01	-0.04	0.03	-0.01	0.04	0.00	-0.03	0.18	0.06
BTACT WL Immediate	0.01	**0.90**	0.04	0.01	-0.01	0.01	0.01	0.01	0.01	-0.01	-0.02
BTACT WL Delayed	0.00	**0.88**	-0.03	0.00	0.00	-0.01	-0.02	0.00	0.00	0.01	0.01
BTACT DB	-0.10	0.18	**0.44**	0.02	0.09	0.03	-0.01	-0.01	0.06	0.10	-0.08
BTACT CF	-0.04	0.08	**0.54**	0.00	-0.05	0.02	0.00	0.00	-0.04	-0.08	-0.02
BTACT NS	0.02	0.02	**0.68**	-0.03	0.05	-0.03	-0.04	-0.01	0.01	0.03	0.06
BTACT BC	0.00	-0.02	**0.78**	0.01	-0.05	-0.01	0.00	0.00	0.00	-0.02	-0.03
Brain Age	0.01	-0.01	0.00	0.02	-0.02	0.01	0.02	0.00	0.00	0.00	**0.99**
Hearing	-0.01	0.06	-0.17	0.18	-0.03	0.04	-0.09	-0.07	0.00	**0.36**	0.18
Balance Area	0.02	-0.01	-0.01	0.15	0.03	0.02	-0.03	0.01	-0.03	**0.59**	-0.07
Balance Length	0.00	-0.01	0.01	-0.03	-0.03	-0.01	0.02	0.01	0.00	**0.99**	0.02
Health Rating	-0.03	-0.06	0.00	0.23	-0.17	0.06	0.01	0.09	0.20	0.02	-0.16
ADL	-0.10	-0.10	-0.11	**0.36**	-0.12	-0.03	0.03	0.06	0.26	-0.01	0.00
BMI	0.28	0.03	0.00	0.10	**-0.71**	0.06	0.06	0.01	0.22	-0.03	-0.04
DHEAS	0.27	-0.02	0.09	-0.06	0.10	-0.01	0.01	-0.02	-0.11	-0.04	-0.25
IGF	0.26	0.04	0.02	-0.06	0.11	-0.01	-0.06	-0.03	-0.20	0.01	-0.23

[a] Values in table are standardized oblimin-rotated loadings from an exploratory factor analysis using maximum likelihood estimation. Loadings greater than or equal to 0.3 in absolute value are shown in bold.

Table S14. Confirmatory Factor Analysis Fit Statistics[a]

	k	-lnL	RMSR	RMSEA	BIC	AIC
**1-Factor**						
Wave 1	75	-79824.72	0.143	0.073	160296.1	159799.4
Wave 2	87	-114390.33	0.121	0.062	229567.0	228954.7
**8/9-Factor**						
Wave 1	99	-77444.48	0.101	0.050	155742.6	155087.0
Wave 2	119	-111145.92	0.099	0.044	223367.4	222529.8
**Bifactor**						
Wave 1	96	-76522.81	0.085	0.034	153873.3	153237.6
Wave 2	112	-109570.66	0.079	0.029	220153.6	219365.3
**9/10-Factor**						
Wave 1	106	-76433.32	0.085	0.033	153780.6	153078.6
Wave 2	127	-109369.82	0.085	0.027	219887.4	218993.6

[a] k = number of model parameters, -lnL = negative log-likelihood, RMSR = root mean square residual, RMSEA = root mean square error of approximation, BIC = Bayesian Information Criterion, AIC= Akaike Information Criterion

Table S15. CFA Loading Estimates[a]

	8/9-Factor		Bifactor			
	Wave 1	Wave 2	Wave 1		Wave 2	
			General	Specific	General	Specific
**Frailty/Strength**						
Grip Strength	0.887	0.843	0.407	0.758	0.368	0.712
Peak Flow	0.756	0.739	0.381	0.683	0.425	0.642
Bone Mineral Density	0.624	0.615	0.189	0.615	0.041	0.715
Waist-Hip Ratio	0.431	0.462	-0.169	0.576	-0.086	0.576
**Cognition**						
BTACT WL Immediate	0.888	0.895	0.390	0.818	0.413	0.798
BTACT WL Delayed	0.870	0.864	0.359	0.801	0.357	0.818
BTACT DB	0.382	0.409	0.715	0.023	0.745	0.019
BTACT CF	0.382	0.403	0.563	0.104	0.573	0.109
BTACT NS	0.381	0.392	0.673	0.044	0.716	0.020
BTACT BC	0.421	0.423	0.423	0.231	0.465	0.204
**Adiposity**						
BMI	0.961	0.797	-0.211	0.64	-0.202	0.682
Lean Mass	-0.492	-0.756	0.294	-0.608	0.193	-0.837
CRP	0.349	0.425	-0.237	0.429	-0.218	0.363
**Glucose**						
Fasting Glucose	0.876	0.832	-0.175	0.844	-0.189	0.799
Hemoglobin A1c	0.876	0.832	-0.293	0.844	-0.286	0.799
**Lipids**						
Triglycerides	0.582	0.438	-0.067	0.579	-0.059	0.444
Cholesterol	0.582	0.438	0.013	0.579	0.141	0.444
**Adaptive Functioning**						
Timed Walk	0.729	0.699	-0.538	0.454	-0.528	0.414
ADL	0.714	0.764	-0.411	0.602	-0.426	0.657
Chair stand	0.682	0.558	-0.564	0.355	-0.437	0.328
Health Rating	0.510	0.557	-0.134	0.557	-0.199	0.543
**Inflammation**						

IL-8	0.456	0.440	-0.115	0.426	-0.217	0.399
TNF-α	0.456	0.440	-0.215	0.426	-0.161	0.399
**Blood Pressure**						
Systolic BP	0.771	0.791	-0.225	0.773	-0.166	0.793
Diastolic BP	0.771	0.791	0.035	0.773	0.021	0.793
**Neurological Functioning**						
Balance Length		0.894			-0.177	0.983
Balance Area		0.634			-0.185	0.549
Hearing		0.478			-0.356	0.354
Brain Age		0.453			-0.317	0.362

[a] Values are standardized maximum-likelihood CFA loadings, from a correlated 8 or 9-factor model, or a bifactor model with uncorrelated factors. Note that the neurological functioning indicators were only assessed in the second wave.

Table S16. Correlations between Aging Factors and Epigenetic Markers: Overall Sample

	Horvath	Hannum	PhenoAge	GrimAge	DunedinPACE
**T1**					
Strength	-0.035	-0.018	-0.050	-0.081	-0.027
Cognition	-0.020	-0.048	-0.063	***-0.148***	**-0.126**
Adiposity	0.040	***0.167***	***0.194***	0.030	***0.315***
Blood Glucose	0.052	-0.001	0.017	0.015	**0.066**
Blood Pressure	-0.045	0.027	0.069	0.020	0.015
Inflammation	0.005	**0.065**	**0.054**	0.038	0.048
Blood lipids	0.017	**-0.084**	-0.061	-0.012	-0.004
Adaptive Functioning	-0.017	-0.050	0.078	***0.166***	***0.157***
**T2**					
Strength	0.058	**0.072**	-0.020	***-0.103***	**-0.078**
Cognition	-0.033	-0.046	**-0.070**	***-0.132***	***-0.107***
Adiposity	0.032	0.068	***0.163***	**0.075**	***0.288***
Blood Glucose	-0.023	0.005	0.031	0.017	***0.136***
Blood Pressure	0.029	0.031	0.058	0.009	0.046
Inflammation	**0.089**	***0.097***	**0.068**	0.038	0.022
Blood lipids	-0.045	-0.031	-0.014	***0.095***	0.012
Adaptive Functioning	0.021	0.021	0.045	***0.185***	***0.168***
Neurological Functioning	-0.062	-0.042	-0.053	0.019	0.016

[a] Values are Pearson correlations; correlations $p < 0.05$ are shown in bold italic; $p < 0.005$ are shown in bold.

Table S17. Prediction of Survival from Aging Factors: Within-Pair Effect[a]

	N*	R^2	β	se(β)	p	q
**Mean**						
Strength	224	0.00	-0.05	0.39	0.91	0.91
Cognition	330	0.01	0.08	0.15	0.61	0.87
Adiposity	337	0.01	0.07	0.15	0.64	0.87
Blood Glucose	221	0.00	-0.05	0.38	0.90	0.91
Blood Pressure	224	0.03	-0.19	0.21	0.38	0.87
Inflammation	221	0.11	-0.33	0.16	0.04	0.28
Blood lipids	221	0.03	0.18	0.38	0.65	0.87
Adaptive Functioning	365	0.05	-0.23	0.13	0.07	0.28
**T1**						
Strength	387	0.03	0.18	0.12	0.15	0.52
Cognition	374	0.00	-0.07	0.13	0.61	0.70
Adiposity	388	0.01	-0.08	0.12	0.51	0.69
Blood Glucose	386	0.01	-0.10	0.13	0.46	0.69
Blood Pressure	387	0.00	-0.02	0.11	0.86	0.86
Inflammation	386	0.02	-0.14	0.08	0.09	0.52
Blood lipids	386	0.02	0.14	0.13	0.26	0.52
Adaptive Functioning	388	0.02	-0.13	0.11	0.25	0.52
**T2**						
Strength	224	0.01	-0.10	0.27	0.72	0.82
Cognition	342	0.11	0.32	0.13	0.01	0.06
Adiposity	337	0.01	0.11	0.15	0.46	0.69
Blood Glucose	221	0.02	0.13	0.53	0.81	0.82
Blood Pressure	224	0.00	-0.05	0.23	0.82	0.82
Inflammation	221	0.18	-0.42	0.19	0.03	0.08
Blood lipids	221	0.13	0.36	0.23	0.13	0.23
Adaptive Functioning	365	0.10	-0.32	0.12	0.01	0.05
Neurological Functioning	85	0.05	-0.23	0.14	0.10	0.23

[a] Values are sample size for the predictor values (N^*; note that the N for the outcome variable is 11875 in all cases), R^2, standardized slope (β), standard error of the slope, p-value, and q-value.

Table S18. Prediction of Survival from Epigenetic Variables: Within-Pair Effect

	N*	R^2	β	se(β)	p	q
Horvath	219	0.00	-0.01	0.21	0.98	0.98
Hannum	219	0.03	-0.16	0.23	0.50	0.74
PhenoAge	219	0.02	-0.13	0.17	0.45	0.74
GrimAge	219	0.15	-0.39	0.12	< 0.001	0.01
DunedinPACE	219	0.01	-0.09	0.19	0.63	0.74
State Factor	219	0.01	-0.11	0.22	0.63	0.74
Change Factor	219	0.06	-0.25	0.15	0.10	0.34

[a] Values are sample size for the predictor values (N*; note that the N for the outcome variable is 11875 in all cases), R^2, standardized slope (β), standard error of the slope, p-value, and q-value.

Table S19. *Prediction of Survival from Aging Factors and Epigenetic Variables: Within-Pair Effect*

	N*	β	se(β)	p	q
**T1**					
Strength	387	0.51	0.16	< 0.001	< 0.001
Cognition	374	-0.54	0.35	0.12	0.22
Adaptive Functioning (-)	388	0.37	0.35	0.29	0.40
Inflammation	386	-0.09	0.08	0.27	0.40
**T2**					
Strength	224	-0.47	1.43	0.74	0.81
Cognition	342	0.70	0.20	< 0.001	< 0.001
Adaptive Functioning (-)	365	-0.37	0.06	< 0.001	< 0.001
Inflammation	221	-0.44	0.14	< 0.001	< 0.001
**Epigenetic Markers**					
PhenoAge	219	0.07	0.20	0.72	0.81
GrimAge	219	-0.35	0.19	0.07	0.15
DunedinPACE	219	0.05	0.69	0.95	0.95

[a] Values are sample size for the predictor values (N*; note that the N for the outcome variable is 11875 in all cases), R^2, standardized slope (β), standard error of the slope, p-value, and q-value.
